# Uniaxial Strain‐Driven Ferroelastic Domain Tuning in ${\mathrm{LaAlO}}_{3}$

**DOI:** 10.1002/advs.78098

**Published:** 2026-10-14

**Authors:** Matthias Roeper, Robin Buschbeck, Jakob Wetzel, Tobias Ritschel, Anna‐Lena Hofmann, Vladyslav Kovtunovych, Mike N. Pionteck, Javier Taboada‐Gutiérrez, Alexey B. Kuzmenko, Martina Basini, Vivek Unikandanunni, Iuliia Kiseleva, Jochen Geck, Susanne C. Kehr, Maximilian Lederer, Simone Sanna, Lukas M. Eng, Samuel D. Seddon

**Affiliations:** ^1^ Institut für Angewandte Physik Technische Universität Dresden Dresden Germany; ^2^ Institute of Solid State and Materials Physics Technische Universität Dresden Dresden Germany; ^3^ Würzburg‐Dresden Cluster of Excellence (EXC2147) ctd.qmat – Complexity Topology, and Dynamics in Quantum Matter Dresden Germany; ^4^ Institut für Theoretische Physik and Center for Materials Research (LaMa/ZfM) Justus‐Liebig‐Universität Gießen Gießen Germany; ^5^ Department of Quantum Matter Physics (DQMP) University of Geneva Geneva Switzerland; ^6^ Physics Department ETH Zurich Zürich Switzerland; ^7^ Institute of Applied Physics University of Bern Bern Switzerland; ^8^ Fraunhofer Institute for Photonic Microsystems IPMS Center Nanoelectronic Technologies (CNT) Dresden Germany

**Keywords:** atomic force microscopy, ferroelasticity, LaAlO3, uniaxial applied strain

## Abstract

Multiferroic domain walls in functional oxides exhibit properties distinct from the bulk and are increasingly exploited as active elements in nanoelectronic and photonic devices. Deterministic tuning of domain populations has typically remained limited to local solutions, or removal with temperature. Here, the manipulation of ferroelastic domain structure, and removal of the twin walls between them in single‐crystal ${\mathrm{LaAlO}}_{3}$ using in situ uniaxial strain is demonstrated. Combining atomic force microscopy, X‐ray diffraction, and Raman spectroscopy with first‐principles calculations, the microscopic evolution of the twin domain population is mapped through a strain‐driven transition from the rhombohedral $R\bar{3}c$ ground state toward the predicted orthorhombic Fmmm phase. Applied strains below 0.5% produce pronounced surface flattening and large‐scale domain reorganization, establishing uniaxial strain as a technically accessible tuning parameter for ferroelastic domain engineering. These results open a route to active alterations of domain architectures in ${\mathrm{LaAlO}}_{3}$‐based heterostructures, with implications for epitaxial film properties, nanoscale phonon‐polariton optics, and ultrafast lattice control.

## Graphical abstract

The present work demonstrates based on AFM, μ‐Raman spectroscopy, XRD, and first‐principles, that uniaxial strain provides a handle for tuning the ferroelastic domain populations in lanthanum aluminate, driving a continuous and controlled phase transition from a four‐variant rhombohedral crystal system to a two‐variant orthorhombic crystal system at strain levels below 0.5% or equivalently ∼ 0.7 GPa applied pressure.

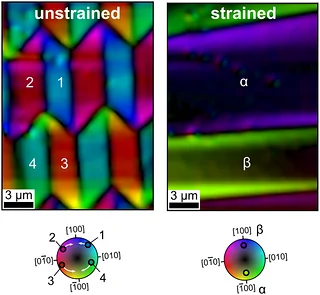

## 1 Introduction

Ferroic materials possess switchable order parameters, such as electric polarization, magnetization, or strain, whose reversible state by conjugate fields underpins a broad spectrum of modern technologies [1, 2]. The boundaries between domains, known as domain walls, have emerged as functional nanoscale objects whose properties differ markedly from the surrounding bulk, opening routes to memristive, synaptic, and other nanoelectronic device concepts [1, 2, 3]. Their high spatial mobility enables real‐time tunability through external stimuli such as electric fields in ferroelectrics [4] or mechanical stress in ferroelastics [5]. Ferroelasticity, the largest ferroic class [6], is characterized by hysteretic switching between two or more crystallographic orientation states under mechanical stress. The spontaneous strain accompanying a ferroelastic phase transition gives rise to complex twin microstructures whose wall geometry and population distribution are intimately coupled to the local strain state [7, 8, 9].

Beyond their role as model systems for symmetry breaking, ferroelastic domains actively mediate the functional response of a wide class of materials: they govern the electromechanical output of piezoelectrics and multiferroics [10], control mechanical dissipation near structural phase transitions [11, 12, 13] with ferroelastic composites enabling extreme damping [14] and serve as targets for nanoscale domain engineering [3, 15]. Stabilized ferroelastic switching further enables non‐volatile magnetoelectric operation relevant to high‐density memories [16], while in inorganic perovskites epitaxial stress at domain walls has been exploited to nucleate unusual two‐dimensional phases with emergent chemical and magnetic order, an effect predicted to be generic to strained orthorhombic $ABO_{3}$ systems [17]. Controlled manipulation of ferroelastic domain populations therefore represents a central challenge, with direct implications for strain‐tunable oxide heterostructures. Whilst some success has been achieved on the local scale [18, 19, 20], global stress/strain tuning has received relatively scant attention. As device miniaturization drives ever‐increasing stress in substrate–film systems, such a handle may enable active domain engineering from tunable superconducting interfaces to atomic‐scale diffusion channels [21].

Lanthanum aluminate (${\mathrm{LaAlO}}_{3}$; LAO), a ferroelastic perovskite oxide, serves as the canonical model system for ferroelastic domain physics [22, 23, 24, 25], exhibiting polar tweed textures that connect to broader questions of hierarchical pattern formation [25]. As a substrate, the LAO domain structure directly governs the properties of overlying thin films, including the critical temperature of superconductors [26], the magnetic anisotropy of ferromagnetic layers [27], and direction‐dependent lattice distortions in ${\mathrm{BiFeO}}_{3}$ [28, 29, 30]. As a constituent layer of the two‐dimensional electron gas at the LAO/${\mathrm{SrTiO}}_{3}$ (STO) heterointerface [31] —which hosts conductivity, superconductivity, and ferromagnetism [32, 33, 34], gate‐tunable through a superconductor–insulator quantum critical point [35] without the chemical disorder of conventional doping [34]— LAO is of particular relevance, as these interface properties are sensitive to the strain state of the LAO layer. Recent demonstrations of resonantly driven infrared‐active [36] and parametrically excited Raman‐active [37] phonon modes further point to ultrafast lattice‐control capabilities, while LAO domain walls show precedent for differing from the bulk, allowing anharmonic coupling between Raman and IR‐Raman modes [38], and as a possible platform for nanoscale light confinement via phonon‐polariton canalization [39]. Below a structural phase‐transition temperature of $544^{\circ }C$, LAO condenses from the undistorted cubic $Pm\bar{3}m$ phase via a soft‐mode‐driven antiferrodistortive transition [40] into the rhombohedral $R\bar{3}c$ space group [7], supporting a rich variety of twin domain patterns [7, 8, 25] with up to four symmetry‐allowed domain states on an (001) surface [7]. Previous efforts to manipulate this domain structure have relied primarily on temperature [41] and hydrostatic pressure, applied to both single crystals [40] and powders at pressures up to 63 GPa [22], as well as on dynamic mechanical experiments [11, 12]. First‐principles calculations predict that the orthorhombic Fmmm phase should become stable under comparatively modest uniaxial stress of ∼1GPa [28], suggesting uniaxial strain as a highly accessible handle for domain reorganization. However, early uniaxial studies were limited to macroscopic characterization of sintered polycrystalline material, where voids and grain boundaries precluded microscopic insight into the intrinsic domain response [42, 43]. Here, this gap is addressed directly, combining scanning probe microscopy, X‐ray diffraction, and Raman spectroscopy with first‐principles calculations to establish a comprehensive microscopic picture of the strain‐driven ferroelastic phase transition in single‐crystal LAO.

## 2 Results and Discussion

### 2.1 Surface Inclination Control

The pseudo‐cubic perovskite representation of the LAO lattice (Figure 1a) highlights the characteristic rotation of the oxygen octahedra about the [111] axis, which drives the rhombohedral distortion from the ideal cubic perovskite structure. Below the ferroelastic phase transition, this symmetry breaking reduces the cubic point group to rhombohedral symmetry, while on the (001) surface four symmetry‐allowed domain variants emerge (see Figure 1b), related to one another by the lost cubic symmetry operations. Each variant is characterized by a distinct in‐plane strain distortion whose orientation reflects the projection of the rhombohedral distortion axis onto the (100) plane. The four variants are not independent: they are grouped into two pairs of twin domains, and the interfaces between them are constrained by compatibility conditions [44] to lie in the (010), (110) and $\left(1\bar{1}0\right)$ planes (for a (001) surface). The collective arrangement of these variants across the surface gives rise to the characteristic chevron twin pattern (Figure 1c), in which domain walls form continuous zig‐zag lines alternating between the two allowed wall orientations and straight lines along the [010] direction. This highly regular pattern is a direct consequence of the ferroelastic nature of the transition and the strict geometric constraints imposed on compatible domain wall orientations in a system with four‐fold surface symmetry. Uniaxial strain, in this work, is applied using the homebuilt piezo‐driven strain cell [45, 46, 47] (Figure 1d), in which stress is transmitted to the sample held between two plates and monitored by calibrated strain gauges.

**FIGURE 1:**
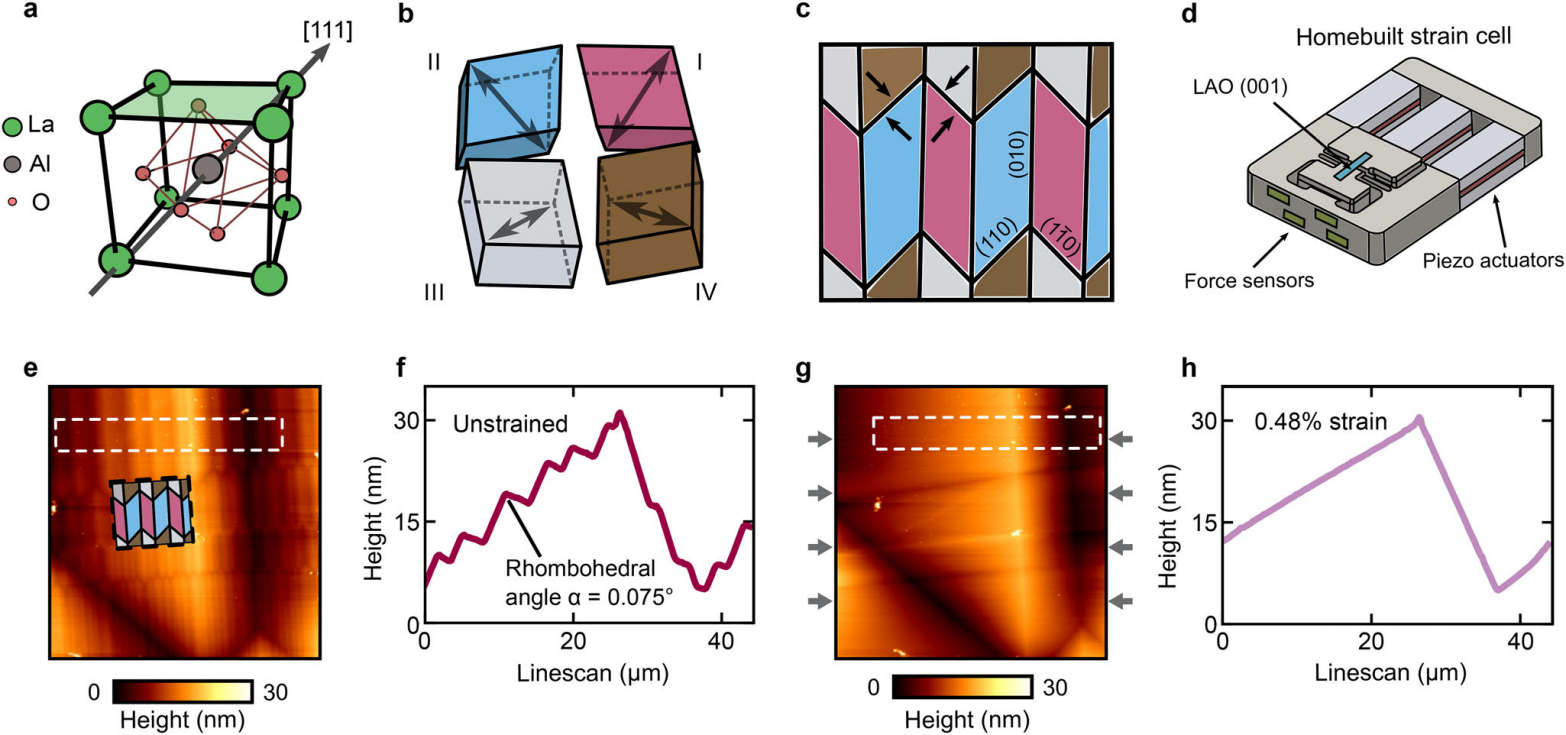
LAO lattice structure, domain formation and strain response. | (a) Pseudo‐cubic LAO lattice with oxygen octahedra (red) slightly rotated about the [111] axis. (b) Four symmetry‐allowed domain orientations on the (001) surface with characteristic strain distortions. (c) Chevron twin pattern on the (001) LAO surface (transparent green surface in a) comprising four domain types separated by walls along the (010), (110) and $\left(1\bar{1}0\right)$ planes. (d) Schematic of the homebuilt piezo‐driven strain cell. (e) $\left(50\times 50\right)\;\mu m^{2}$ AFM height scan revealing ferroelastic micro‐domains (with a few micrometer periodicity) superimposed by non‐ferroelastic structures (periodicities of a few tens of micrometers). (f) Vertically averaged line scans (white dashed region in (e) showing inclined surface normals forming a stepped structure. (g) AFM scan under 0.48% applied compressive strain (strain axis indicated by gray arrows) showing a strongly reduced surface substructure. (h) AFM height line scan under strain demonstrating surface flattening relative to (f).

The AFM topography image acquired under ambient conditions (Figure 1e) resolves this domain structure in detail. Ferroelastic domains with periodicities of a few μm in width are clearly present across the surface. Importantly, these are superimposed by larger surface undulations with periodicities of few tens of μm, which show self‐similarity to the walls present in the microstructure, but are a real surface topography rather than ferroelastic domain structure. This scale invariant effect present in ferroelastics is already well established, and is the result of repeated polishing and annealing [8]. Vertically averaged line scans extracted from the white dashed region (Figure 1f) confirm that neighboring ferroelastic domains present distinctly inclined surface normals, forming a stepped profile whose amplitude directly reflects the magnitude of the rhombohedral distortion. This careful distinction between these two classes of surface structure, i.e., between actual ferroelastic domains and pure topographic features, is essential for the unambiguous interpretation of strain‐dependent scanning probe data presented below.

Upon the application of 0.7 GPa uniaxial compressive stress, the surface morphology undergoes a dramatic transformation (Figure 1g), with the ferroelastic domain structure visible under ambient conditions strongly suppressed, while the larger‐scale non‐ferroelastic topographic features persist as expected. Consistent with this, the stepped surface profile observed in Figure 1f flattens considerably under strain (Figure 1h), reflecting a near‐complete redistribution of the ferroelastic domain population. Such removal of the surface facets is directly relevant to thin films grown on top, which have been shown to dominate epitaxial thin film properties grown on LAO. It should be noted, however, that any strain applied in this method to a thin‐film / LAO substrate system, would not only transfer via changes to the LAO domain structure (and epitaxy), but also from the strain cell directly compressing the film itself too [45], making the decoupling of measured effects non‐trivial.

This observed facet removal behavior is indicative of a strain‐driven selective stabilization of domain variants favorably aligned with the applied uniaxial strain direction, at the expense of unfavorably oriented variants. The fact that this redistribution occurs at moderate strain levels suggests that the energy barrier separating competing domain orientations in LAO, is small, rendering the ferroelastic domain structure highly susceptible to external mechanical perturbation. Closer examination of the two AFM images reveals the presence of some faint ferroelastic domains, extending across the entire sample horizontally (see Figure 3$a_{4}$ / Figure S2b, being still present under applied strain, implying that in fact, whilst reoriented, there are still some twin walls and therefore structural difference between horizontal pairs of domains.

### 2.2 Advanced Structural Analysis

In order to further analyze the remaining domain structure under applied strain, the AFM topography images were differentiated along the two orthogonal in‐plane directions to create a local map of surface normal. The doubly differentiated AFM image acquired under ambient conditions can be seen in Figure 2a, with inclination direction and amplitude color‐coded for clarity (Figure 2b). Four distinct domain types are resolved, consistent with the symmetry‐allowed variants anticipated from Figure 1c and labeled I–IV, whose corresponding rhombohedral distortions are illustrated in Figure 2b. Here, the color scale can be interpreted as the in‐plane direction, with each surface normal pointing out of the page. The presence of all four variants (i.e., as in the schematic Figure 2c) is independently confirmed by 3D‐XRD reciprocal space mapping. Imaging the (002) specular Bragg reflection along all three Q‐space directions allows for the projection of the splitted peak onto the $Q_{\left[100\right]}$–$Q_{\left[010\right]}$ plane (Figure 2d), which reveals four well‐separated sub‐peaks whose positions are fully consistent with the four domain orientations identified by AFM. The combined real‐ and reciprocal‐space characterization thus establishes an unambiguous correspondence between the surface domain structure and the underlying bulk lattice distortions. From the angle between each stepped terrace (0.075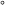 at room temperature) follows directly the rhombohedral crystallographic angle of $\alpha^{\prime }=90^{\circ }+\alpha$. This is in good agreement with the averaged surface tilt determined via the XRD measurements of $\Delta Q_{∥}\cdot Q_{⊥}^{-1}=0.091^{\circ }$, and consistent with previous literature values such as Bueble et al. (0.096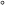) [8].

**FIGURE 2:**
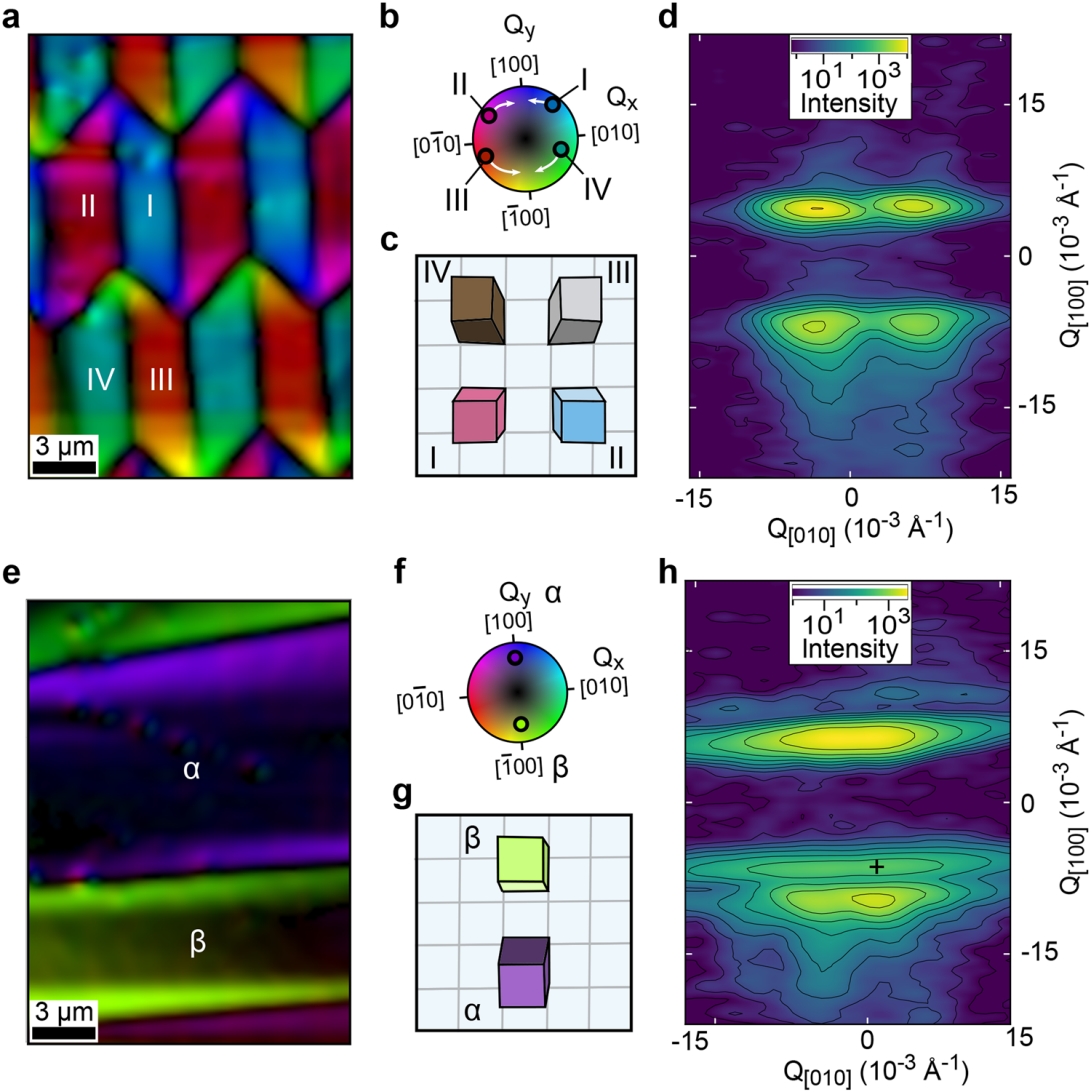
Structural transition probed by AFM and XRD | (a) Doubly differentiated AFM image (effectively mapping the surface normal) under ambient conditions where surface inclination direction and amplitude are colour‐coded as indicated in (b). Four domain types are present as expected from Figure 1c, labeled I–IV. (b) Inclination directions (I–IV) extracted from the AFM scan, with the corresponding rhombohedral distortions shown exaggerated in (c). (d) 3D‐XRD measurement projected onto the $Q_{\left[100\right]}$–$Q_{\left[010\right]}$ plane (in‐plane directions), showing four peaks consistent with the four domain types identified by AFM. (e) Doubly differentiated AFM scan at the same position as a under applied stress of 0.7 GPa. Domains I and III vanish with increasing strain while domains II and IV extend over the entire scan area. (f) Under strain, the inclinations of the two remaining domain types (denoted as α and β) are oriented along the [001] axis, as expected of two orthorhombic domains (g). (h) 3D‐XRD projection under maximal strain; the four peaks merge into two, as well as the emergence of a third peak indicated with a black '+' right above the main peak, which originates from needle domains, and is further discussed in the Figure S2.

Upon application of uniaxial strain along the [010] axis, the domain structure undergoes a clear and reproducible reconfiguration. The equivalent image acquired under strain (Figure 2e) shows domains I and III progressively vanish with increasing strain (also demonstrated for a larger area in Figure 3a_1_–a_5_), while domains II and IV expand to cover the entire scan area (hereby labeled α and β). The two surviving, transformed domain types retain inclinations oriented along the [010] direction (Figure 2f), consistent with the expected orthorhombic distortion geometry of a two‐variant ferroelastic state (Figure 2g). Figure 2f shows the effect of these surface tilts on the in‐plane color map, with a strong surface tilt still present between domains, as reflected in schematic Figure 2g.

**FIGURE 3:**
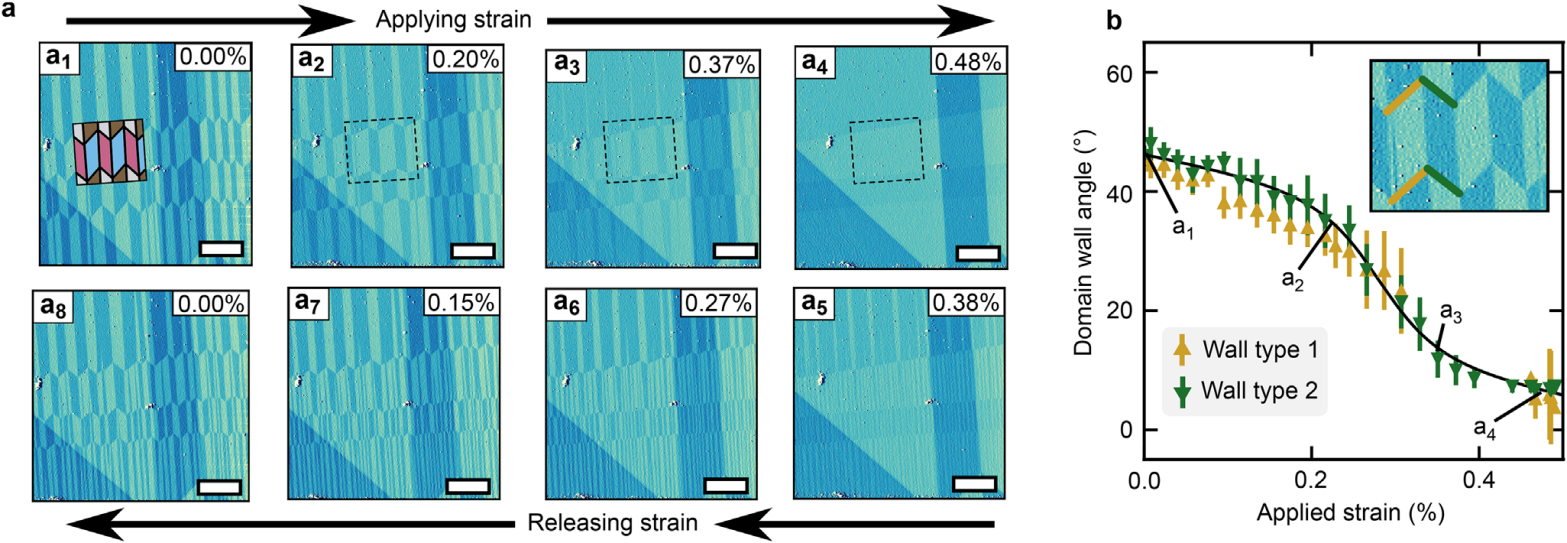
In‐plane twin wall angle rotation | (a) Full strain cycle applying (a1–**a4**) and releasing (a5–a8) compressive strain, along the horizontal axis, up to 0.48% (∼0.7 GPa stress) captured by AFM error signal scans (scale‐bar 10 μm). (a1) Domain walls form zig‐zag lines inclined at $45^{\circ }$ as expected from Figure 1c. With increasing strain the angle decreases continuously until the walls flatten to $0^{\circ }$ (a4), accompanied by one domain type progressively consuming the surface area of the other. (a5–a8) The process is fully reversible: upon strain release the domain wall angle increases and the surface share of the receding domain type recovers. (b) In‐plane domain wall angle with respect to the (010) plane as a function of applied strain, extracted from the dashed region in (a1–a4), analyzed independently for the two domain wall orientations (yellow and green), a line (black) is a guide to the eye. The domain wall angle evolves continuously with strain.

This strain‐driven symmetry reduction from a four‐variant to a two‐variant domain population is also confirmed in reciprocal space: the 3D‐XRD projection acquired at maximum applied strain (Figure 2h) shows that the four peaks present under ambient conditions merge into two, directly reflecting the suppression of the unfavorably oriented domain variants. Given that a (100) twin wall is energetically not preferred [7, 8] in this crystal setting, such an increase in symmetry despite a lasting presence of distinct domains, is fully consistent with that of an orthorhombic Fmmm structure. Indeed this was previously predicted by DFT calculations of uniaxial strain by Hatt and Spaldin [28] resulting in a stable orthorhombic structure, though no discussion as to domains therein were addressed.

The central result of this combined AFM and XRD analysis is that uniaxial strain drives a controlled, reversible transition from a four‐variant to a two‐variant ferroelastic domain state, and also a rhombohedral $R\bar{3}c$ – orthorhombic Fmmm phase transition, with the domain redistribution manifesting simultaneously and consistently in both real and reciprocal space.

### 2.3 Continuous Twin Wall Rotation

The AFM error signal can be interpreted as a fast‐scan axis derivative, and is therefore clearly correlated to topographic features. AFM error signal scans acquired across a full strain cycle (Figure 3a_1_–a_8_) provide a detailed real‐space picture of the domain wall dynamics underlying the rhombohedral to orthorhombic phase transition already established. Applied strain values were acquired from in‐plane lattice parameter shift in XRD measurements (see supplement F). Under ambient conditions (Figure 3a_1_), domain walls form continuous zig‐zag lines inclined at $∼45^{\circ }$ to the strain axis, consistent with the chevron pattern expected from the four‐fold domain symmetry in LAO [8]. As uniaxial strain is applied, this angle decreases continuously and monotonically, with the domain walls rotating progressively toward alignment along the strain axis until they flatten to $0^{\circ }$ at maximum applied strain (Figure 3a_4_). This angular evolution is accompanied by a simultaneous redistribution of domain surface area: within each horizontal line of the scan, one domain type progressively expands at the expense of the other, until the minority variant is fully consumed at maximum strain. Together, the wall rotation and the surface area redistribution represent two complementary manifestations of the same underlying strain‐driven domain repopulation process. Both processes are fully reversible: Upon release of the applied strain (Figure 3a_5_–a_8_), the domain wall angle increases again and the surface share of the previously suppressed domain type recovers, with the surface returning to the ambient $45^{\circ }$ chevron configuration (for several repeated cycles see Video S1). As discussed, a small amount of hysteresis is seen, as even in zero field needle domains continue to propagate throughout the sample, however given sufficient relaxation (τ = 4.8 h see Figure S1) Figure 3$a_{8}$ returns to the equivalent domain state as Figure 3a_1_. Although immediately at zero strain the twin wall angle has returned to its original angle, the domain state still requires some time to fully relax (as typical with many other magnetic or ferroelectric switching regimes). In order to switch all functional parameters close to real‐time, either the given hysteresis is for a potential application sufficiently small or a sample with fewer defects has to be chosen to further suppress a nucleation of spike domains [48, 49, 50].

To quantify the wall rotation, the in‐plane domain wall angle was extracted as a function of applied strain independently for the two domain wall orientations present on the surface, initially oriented along the (110) and ($\bar{1}10$) axes and denoted as wall type one and two (Figure 3b, red and purple), within the region indicated by the dashed box in Figure 3a_1_–a_4_. Both orientations exhibit a continuous, monotonic decrease with increasing strain. The continuity of this angular evolution, with no evidence of an abrupt transition or threshold behavior typical of Barkhausen noise is somewhat unexpected, and nevertheless implies a smooth, second‐order phase transition between the two crystal symmetries.

### 2.4 Lifting of Phonon Degeneracy

LAO is an improper ferroelastic, its order parameter is in fact not strain directly (though strain and the order parameter are directionally coupled) but the octahedral tilt angle [51]. In order to probe the order parameter of this system most directly, this mode must be directly visualized. The first three Raman active modes are a $E_{g}$ mode, a symmetric non‐degenerate $A_{1g}$ mode and a second higher energy degenerate $E_{g}$ mode. These are respectively characterized by oxygen octahedra rotation, La atom oscillation along the [111] axis, and La atom oscillation within a pseudo‐cubic plane. A schematic of these three modes can be found in Figure 4a. All three modes are clearly resolved in the ambient backward‐scattered Raman spectrum in Figure 4b, with peak positions in good agreement with the specifically performed first‐principles predictions, providing a firm spectroscopic baseline against which strain‐induced shifts can be assessed.

**FIGURE 4:**
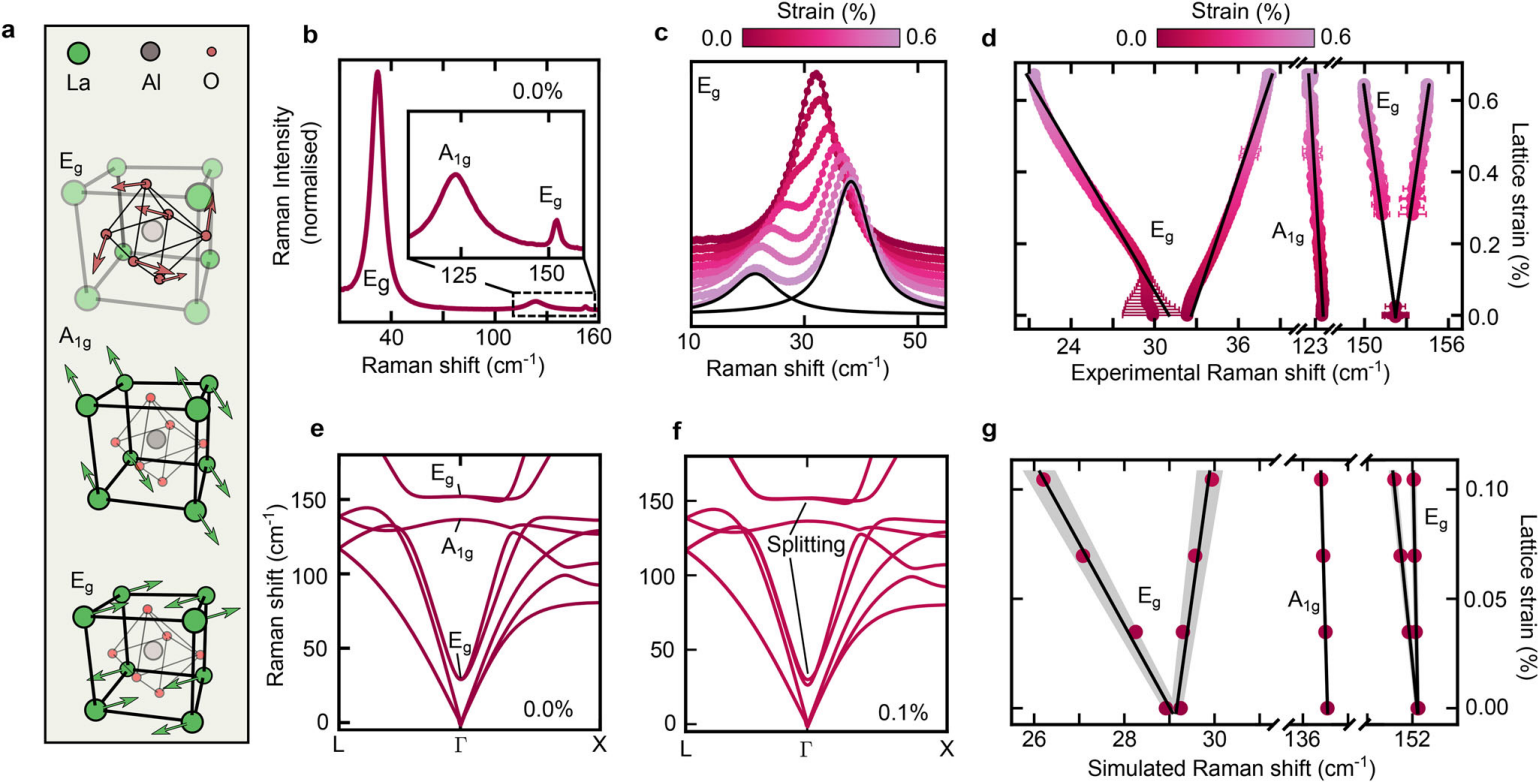
Strain‐dependent phonon behaviour of LAO | (a) Three major modes in LAO corresponding to two $E_{g}$ modes and one $A_{1g}$ mode energetically between them. (b) Typical Raman spectrum of LAO (100) under ambient conditions showing three peaks. (c) Strain‐dependent Raman spectra in the vicinity of the most intense $E_{g}$ mode displaying a strain‐induced lifting of degeneracy (data fitted with a pair of Voigt profiles in black). (d) $E_{g}$ peak splitting and $A_{1g}$ peak softening as a function of strain, extracted from peak fits and extracted values linearly fitted (black). (e) First‐principles phonon dispersion of unstrained LAO near the Γ‐point. (f) Phonon dispersion under 0.1% strain, showing lifting of $E_{g}$ degeneracy at the Γ‐point. (g) Modal behavior of calculations performed across several strain levels between 0% and 0.1% confirming the behaviors for both the $E_{g}$ splitting and the $A_{1g}$ mode softening, as indicated by linear fits (black).

The strain dependence of the phonon spectrum is best displayed in the behavior of the highest intensity $E_{g}$ mode (Figure 4c). Under applied uniaxial strain, the single ambient $E_{g}$ peak progressively splits into two resolved components, directly evidencing a strain‐driven lifting of the rotational degeneracy of the oxygen octahedra vibrations. This splitting is fully consistent with the symmetry reduction from a rhombohedral to a orthorhombic phase, with the application of uniaxial strain breaking the equivalence of the two $E_{g}$ sub‐modes by selecting a preferred distortion axis, rendering them spectroscopically distinct. Systematic peak fitting across all strain levels (Figure 4d) confirms that the $E_{g}$ splitting increases for both degenerate modes approximately linearly with applied strain, while the $A_{1g}$ mode exhibits a continuous softening, consistent with hydrostatic studies [22, 40].

Density functional perturbation theory (DFPT) calculations allow the behavior under uniaxial strain to be further explored. Here, the phonon band structure around the Γ‐point reveals three lowest energy Raman‐active phonon modes (Figure 4e) as measured in Figure 4b. By deforming the relaxed unit cell, based on the calculated compliance tensor, according to a pressure acting along the pseudo‐cubic [100] direction (the same direction as in the experiments), and therefore simulating the effect of applied stress, a visible splitting can be seen in the maximal simulated case (0.1% strain, Figure 4f) at the bands around the Γ‐point. By plotting each mode as before, the relative behaviors as a function of intermediate values of strain reveal a similar lift of degeneracy of the $E_{g}$ modes. A broadly linear splitting is achieved as well as a phonon softening of the $A_{1g}$ mode.

## 3 Conclusion

The present work demonstrates that uniaxial strain provides a highly accessible handle for engineering the ferroelastic domain populations in LAO, driving a continuous and controlled transition from a four‐variant rhombohedral crystal system to a two‐variant orthorhombic crystal system at strain levels below 0.5% or equivalently ∼ 0.7 GPa applied pressure. This transition is met by the complete removal of step terraces in the system along the strain axis, a fact of huge relevance to communities working on LAO systems as an epitaxial substrate. The crystallographic phase transition is further verified by accompanying strain‐induced lifting of the $E_{g}$ phonon degeneracy, confirmed by both Raman spectroscopy and first‐principles calculations. This provides a phonon‐level fingerprint of the symmetry reduction underlying this transition, and establishes a quantitative spectroscopic measure of the crystal state that could be exploited for non‐contact characterization in device geometries moving forwards. The continuous tunability of the domain wall angle raises the prospect of exploiting the LAO domain structure as an active degree of freedom in substrate‐film systems: if the strain‐driven domain redistribution can be shown to couple directly to the transport or magnetic properties of an overlying functional film, this would establish a practical route to in situ tunable oxide devices. More generally, the methodology demonstrated here, combining quantitative atomic force microscopy, three dimensional reciprocal space mapping, and Raman spectroscopy under in situ uniaxial strain, is directly transferable to the wider class of ferroelastic perovskite oxides, many of which share analogous twin domain structures and phase diagrams. Applied to systems where ferroelastic order is coupled to ferroelectric, magnetic, or orbital degrees of freedom, uniaxial strain could allow for the controlled manipulation of functional properties through domain engineering.

## 4 Experimental Section

### 4.1 Lanthanum Aluminate Sample And Implementation In Strain Cell

Double sided polished LAO samples, with (001) orientation, from CRYSTAL GmbH, grown via the Czochralski method, are cut and further polished to obtain bars with the dimensions of ∼(5×0.3×0.2) ${\mathrm{mm}}^{3}$. During the post‐industry cutting and polishing of the backside of the sample the investigated surface is covered by a solvable protection layer to avoid surface defects. These bars are glued with epoxy‐resin onto a piezo‐actuator driven titanium strain cells with force calibrated strain gauges (see Figure 1d), which are readout by a Stanford Research Systems SR830 DSP lock‐in amplifier. The methods of constructing and calibrating the home‐made strain cells as well as the gluing with an ensured force transmission into the sample are well established and proven [45, 46, 47]. The sample is mounted in a way that it is strained along the [010] axis. Knowing the cross section of the sample in combination with a continuous force measurement enables the determination of stress on the corresponding sample during the measurement. To investigate domain properties of the sample under strain a voltage is applied to the piezo‐actuators, which is continuously either ramped up or down. In order to ensure at any point of straining a state which is sufficiently close to adiabatic, the ramping till the maximum strain takes typically two hours. Artifacts from e.g. hysteresis effects are suppressed by cycling the sample on the strain cell several times between the minimal and maximal strain before the actual measurements.

### 4.2 Atomic Force Microscopy (AFM)

The topography measurements are performed on an well established combination [47, 52] of Park NX10 SFM and solid platinum tips 25Pt300B manufactured by Rocky Mountain Nanotechnology, LLC, in contact mode. Typical scanning parameters are (256×256) Pixels with a 1.5 Hz scanning rate along the fast‐scanning axis. The set‐point used, of ∼700 nN, is sufficiently low to ensure that its influence on the dynamics and structures of interest is negligible. The trace and back‐trace of the height and error signal are both recorded. If only the slope along one axis is of interest, the error signal is further analyzed. Since the error signal is the deviation of the PID‐loop the AFM‐height controller is faced [53], it is a measure of the local inclination of the sample surface, while a line alignment along the slow‐scanning axis is intrinsically not required. In case the full two dimensional inclination has to be considered, the multi‐dimensional Jacobian matrix is derived from the height signal. This distinction is done, because the error signal is slightly less noisy and avoids artifacts along the slow‐scanning axis.

### 4.3 μ‐Raman Spectroscopy

The μ‐Raman spectroscopy measurements are acquired on a HORIBA LabRAM HR Evolution system, utilizing a 532 nm, 100 mW continuous wave laser in combination with a 100× magnification objective with a numerical aperture of 0.9 in a back‐scattering geometry. The signal is detected by a Syncerity CCD camera (HORIBA Jobin Yvon GmbH) in combination with a 1800 lines/mm grating, which leads to a spectral resolution of 0.013 nm or 0.48–0.68 ${\mathrm{cm}}^{-1}$, respectively. To determine the peak splitting and softening with Raman spectroscopy, continuous measurements are performed, like the AFM measurements, while the strain is ramped up or down. Besides these comparatively fast measurements, which as a tradeoff cannot resolve the whole sample microscopically, maps with a resolution down to 0.5 μm are performed. Due to an integration time of 0.5 s per pixel with up to (252×252) pixels, the ramping of the strain is stopped at certain levels to allow for longer measurement times. To determine the peak positions from the acquired Raman spectra the background including cosmic ray peaks are removed and a multi Voigt‐fit [54] is performed.

### 4.4 Density Functional Perturbation Theory (DFPT)

For the numerical determination of the phonon dispersion and the corresponding spacial atomic vibrations by density functional perturbation theory [55, 56], implemented in ABINIT [57, 58] and Quantum Espresso [59, 60] are used. Scalar relativistic optimized norm‐conserving Vanderbilt pseudo potentials [61] with Perdew–Burke‐Ernzerhof exchange‐correlation functionals are provided by Pseudo Dojo [62]. The initial experimental crystal structure was selected from SpringerMaterials determined on a powder sample by an automatic neutron time‐of‐flight diffractometer [63]. This structure is then relaxed numerically.

The workflow to determine the atomic vibrations is set via AbiPy. After a converged relaxation (with a force convergence criterium of $10^{-5}\;Ry\;\;a_{0}^{-1}$), the dynamical matrix of the system is calculated, based on a (4×4×4) k‐point grid. The cutoff energy is set to 50 Ha.

In order to determine the strain induced shifts of the Raman peaks the energies at the Γ‐point as well as the phonon dispersion for the maximum and minimum strain are calculated via Quantum Espresso, with a force convergence criterium of $10^{-4}\;Ry\;\;a_{0}^{-1}$. Linear Poisson effects are taken into account by the first principle compliance tensor. The compliance tensor is acquired by systematically analyzing strain–stress tensor pairs of strain perturbed unit cells, while taking the symmetries of the system into account to avoid duplicated computational effort. The same computational parameters, as used beforehand, are used to model the strained unit cells.

### 4.5 X‐Ray Diffraction

For the XRD measurement in the supplement, a Bruker Discover D8 with 1.6 kW Cu‐Ka1 radiation with a wavelength of 1.54 Å has been used with a parallel‐beam geometry. After the Göbel mirror, the slits on the primary side are 0.2 mm and 6×2 mm. No additional optics are present on the secondary side. The detector is the Lynxeye XE‐T. The tool has been controlled by the software DiffracMeasurementCenter and within this, the RSM has been performed with the WIZARD plug‐in.

The three‐dimensional reciprocal space mapping (3D‐RSM) is performed using a custom‐built six‐circle diffractometer. The diffractometer is equipped with an Excillum liquid MetalJet source operating with a Ga/In alloy target. A custom multilayer mirror X‐ray optic selects the In Kα doublet (0.512 Åand 0.517 Å) and focuses the beam to a spot size of 70×50 $\mu m^{2}$ with a beam divergence of 1.5 mrad. Diffracted intensity is recorded in reflection geometry using a Dectris PILATUS3 X CdTe 1M hybrid photon‐counting area detector. Data acquisition, angular‐to‐reciprocal‐space coordinate transformation, and subsequent analysis are carried out using custom Python scripts built on the open‐source packages xrayutilities  [64].

## Conflicts of Interest

The authors declare no conflicts of interest.

## Supporting information


**Supporting File 1**: advs78098‐sup‐0001‐SuppMat.pdf.

**Supporting File 2**: advs78098‐sup‐0001‐SuppVideo.mp4

## Data Availability

The data that support the findings of this study are available from the corresponding author upon reasonable request.
