# Supplementary material for: Uniaxial Strain‐Driven Ferroelastic Domain Tuning in LaAlO3
Source: Adv Sci (Weinh). 2026 Oct 14:e78098. Online ahead of print. doi: 10.1002/advs.78098 (PMC13669226; doi:10.1002/advs.78098)
Supplement: Supplementary file 1 [file ADVS-9999-e78098-s002.pdf]

Supporting Information for: Uniaxial strain-driven ferroelastic domain tuning in $LaAlO_3$

*Matthias Roeper Robin Buschbeck Jakob Wetzel Tobias Ritschel Anna-Lena Hofmann Vladyslav Kovtunovych Mike N. Pionteck Javier Taboada-Gutiérrez Alexey B. Kuzmenko Martina Basini Vivek Unikandanunni Iuliia Kiseleva Jochen Geck Susanne C. Kehr Maximilian Lederer Simone Sanna Lukas M. Eng Samuel D. Seddon**

1 Interpretation of Raman peak splitting

The observed splitting of the E_g modes originates from the fact that the under unstrained conditions degenerated E_g modes are associate with energetically equivalent, but different lattice vibrations (only one vibration pattern is shown per degenerated pair in Fig. 4a), while the vibration of the A_{1g} mode would, applying the same discrete symmetry operation (rotation around the (111) axis by $\pi/2$), end up in itself.

2 Relaxation Of Lanthanum Aluminate Domains After Stress Removal

Since the stress is ramped up to its maximum in a time regime of hours, the domain state of the domains within the sample is considered to be to first order quasi-static. In order to quantify higher order deviations AFM measurements at zero strain where carried out after cycling the stress of the sample several times (Fig. S1). Each AFM scan is performed within a time of a few minutes. Relaxation effects, quantified from a higher order spacial deviation (domain measure Fig. S1a), are measurable even several hours afterwards ($\tau = 4.8$ h) and remaining sufficiently small to consider them as negligible for the performed measurements of the paper. However for higher cycle rates these effects becoming relevant. These spike domains present immediately after stress cycling, are also affecting in the XRD measurements (Fig. S2). This relaxation in zero strain allows furthermore the identification of the lower intensity shoulder peak in Fig. 2h, originating from the still active needle points remaining, and seemingly not linked to the domains themselves of the low strain regime. Constant AFM imaging of the measurement area indicated the motion of needle domains still moving throughout the sample (see supplement Fig. S1), with their eventual stability broadly correlated with the removal of the shouldered XRD-peak seen in Fig. S2g. These needle dynamics are subject to ongoing investigation.

3 Correlation of stress and strain

Since the strain cell is equipped with sensors which are sensitive to the force applied to the sample, the stress can be, directly determined once knowing the samples cross section. In order to make statements with respect to the strain of the sample, XRD measurements of the sample mounted on the strain cell are performed, while ramping up and down the stress. The strain can be immediately calculated from the XRD based reciprocal lattice parameter Q_x. Since the dependence between stress and strain is sufficiently linear, a conversion factor is used whenever quantitative statements with respect to the strain are required. It has to be noted that while the statistical deviations remain small in the order of magnitude of 1% or smaller, systematic deviations are accumulated from the conversion of the lock-in amplitude into a force, followed by the conversion into a stress value and finally into a strain value, resulting in a relative systematic deviation of not less than several 10%. The uncertainty is estimated in a quite conservative approach including the experience from previous studies with the same strain setup (https://doi.org/10.1103/PhysRevB.111.064109). The systematic uncertainty can be further reduced by a full 3D-scan of the sample to determine the cross section more accurate, a better shielding of the force sensor wires, and a compensation of the long-term drift of the force measurement.

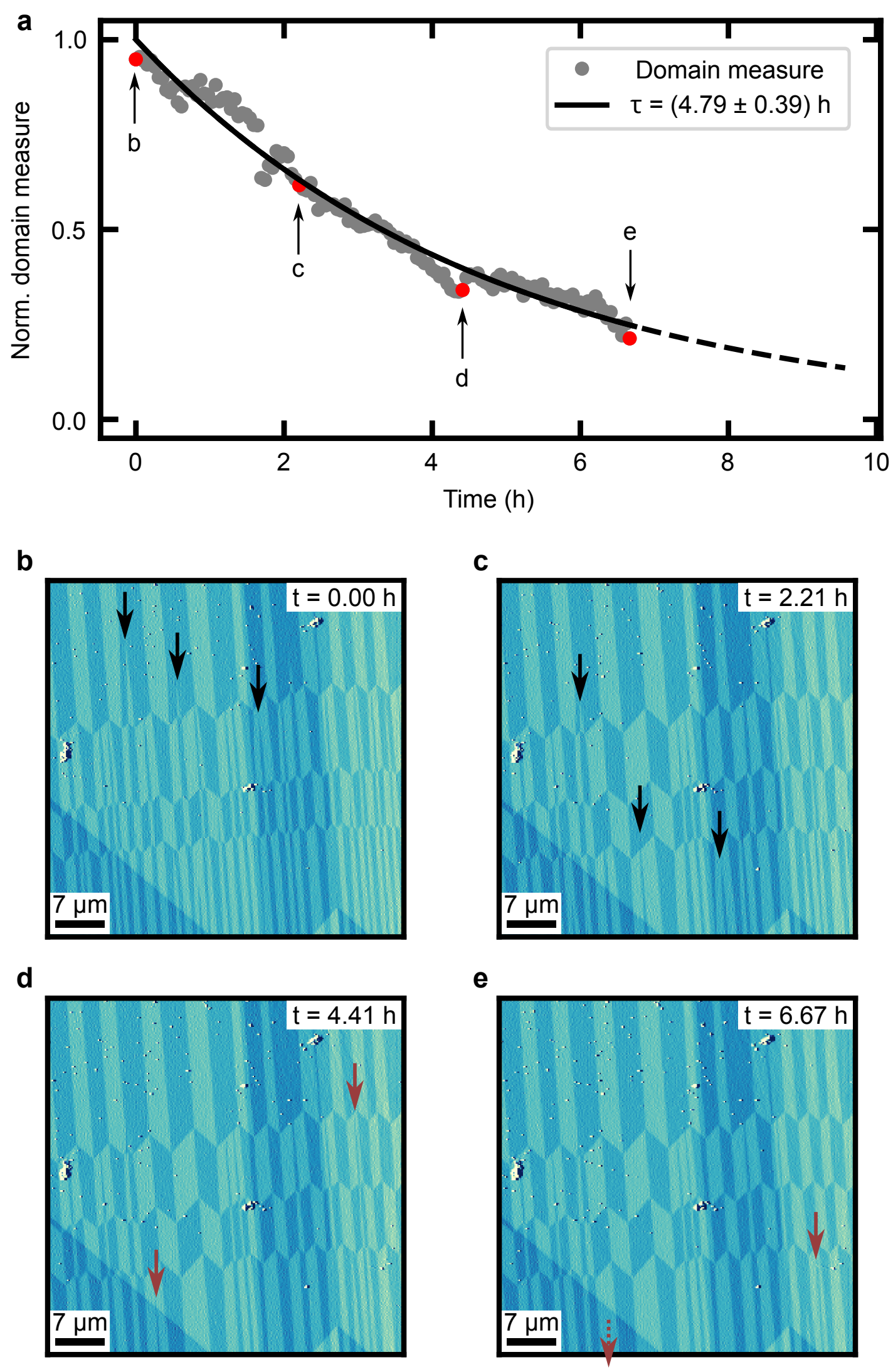

Figure S1: Domain relaxation captured by repeated AFM error signal measurements at different times. **a** Fit of the exponential decay of the domain activity after the external stress is released. **b** Measurement directly after the external stress is released. **e** Almost seven hours afterwards with intermediate measurements (**c**, **d**). Spike domains are still retracting from the upper part of the scan area towards the lower part under zero external stress. Selected spike domains retracting in (b-c) highlighted by black arrows and similarly for (d-e) indicated by red arrows.

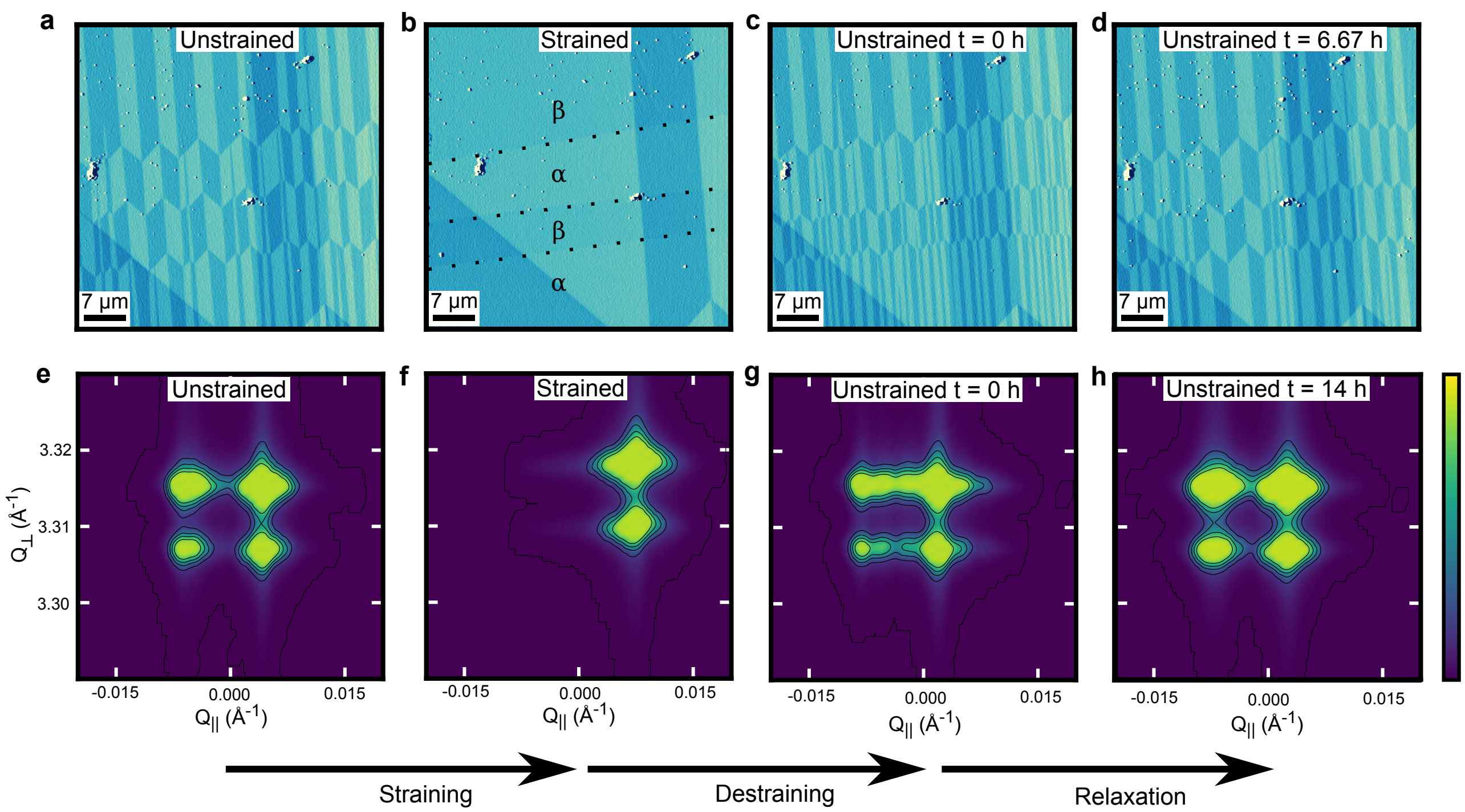

Figure S2: Strain cycling and relaxation qualified by the AFM error signal (**a-d**) and XRD intensity (**e-h**). **a** AFM measurement of the initial unstrained sample with four domain types. **b** Sample at maximum strain with two faintly visible domain types α and β. **c** Destrained sample after several strain cycles with four types of domains, but compared to **a** with additional spike domains. **d** Unstrained sample after 6.67 h of relaxation. Almost all of the additional domains disappeared from the scanned area. **e-h** XRD measurement of an entirely relaxed, unstrained sample, under maximal strain, immediately at the point of zero applied strain, and after 14 hours of relaxation. Note the projection here includes vertical peak splitting due to the Kα doublet, unlike the in-plane projection in the main text. Each reciprocal space map is analogue to the AFM image above it.
